# Effective Improvement of the Photovoltaic Performance of Carbon-Based Perovskite Solar Cells by Additional Solvents

**DOI:** 10.1007/s40820-016-0094-4

**Published:** 2016-05-31

**Authors:** Chenxi Zhang, Yudan Luo, Xiaohong Chen, Yiwei Chen, Zhuo Sun, Sumei Huang

**Affiliations:** Engineering Research Center for Nanophotonics & Advanced Instrument, Ministry of Education, Department of Physics, East China Normal University, Shanghai, 200062 People’s Republic of China

**Keywords:** Halide perovskite, Solar cell, Spin-coating, Carbon counter electrode, Free hole transporting material

## Abstract

**Abstract:**

A solvent-assisted methodology has been developed to synthesize CH_3_NH_3_PbI_3_ perovskite absorber layers. It involved the use of a mixed solvent of CH_3_NH_3_I, PbI_2_, γ-butyrolactone, and dimethyl sulfoxide (DMSO) followed by the addition of chlorobenzene (CB). The method produced ultra-flat and dense perovskite capping layers atop mesoporous TiO_2_ films, enabling a remarkable improvement in the performance of free hole transport material (HTM) carbon electrode-based perovskite solar cells (PSCs). Toluene (TO) was also studied as an additional solvent for comparison. At the annealing temperature of 100 °C, the fabricated HTM-free PSCs based on drop-casting CB demonstrated power conversion efficiency (PCE) of 9.73 %, which is 36 and 71 % higher than those fabricated from the perovskite films using TO or without adding an extra solvent, respectively. The interaction between the PbI_2_–DMSO–CH_3_NH_3_I intermediate phase and the additional solvent was discussed. Furthermore, the influence of the annealing temperature on the absorber film formation, morphology, and crystalline structure was investigated and correlated with the photovoltaic performance. Highly efficient, simple, and stable HTM-free solar cells with a PCE of 11.44 % were prepared utilizing the optimum perovskite absorbers annealed at 120 °C.

**Graphical Abstract:**

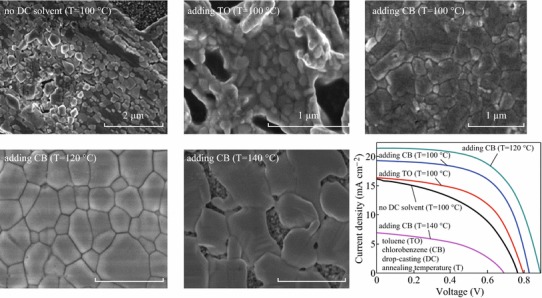

## Introduction

During the past 5 years, there has been a surging interest in the study of organic–inorganic hybrid perovskite compounds for applications in photovoltaic devices because of low cost, simple fabrication process, and high-efficiency solar power conversion [1–6]. Typically, perovskite solar cells (PSCs) employed a mesoporous titania or alumina scaffold, a methylammonium (MA) lead iodide perovskite light absorber, an organic hole transport material (HTM), characteristically spiro-OMeTAD (2,2′,7,7′-tetrakis-(*N*,*N*-di-*p*-methoxyphenylamine)-9,9′-bifluorene), and an Au or Ag electrode. PSCs achieved a power conversion efficiency (PCE) of >10 % in 2012 [2, 3]. TiO_2_/spiro-OMeTAD is regarded as a successful couple owing to its good optical transparency and perfect band alignment with respect to CH_3_NH_3_PbI_3_. Later, the PCEs were improved to 15 % using a two-step sequential deposition technique, involving spin-coating of a PbI_2_ followed by exposure to a solution of CH_3_NH_3_I to form CH_3_NH_3_PbI_3_, or a dual-source vapor deposition technique to fabricate a planar heterojunction solar cell [4, 5]. Then, a planar structured PSC using a polyethyleneimine ethoxylated modified ITO electrode, yttrium-doped TiO_2_ layer, mixed halide perovskite CH_3_NH_3_PbI_3−*x*_Cl_*x*_ absorber, and spiro-OMeTAD reached a PCE of 19.3 % [6]. Very recently, triple Cs/MA/formamidinium cation PSCs reached a power output of 21.1 % [7].

Currently, the most research PSC devices employ gold as a back contact, in conjunction with organic hole conductors acting as electron-blocking or hole transport layers [4–7]. These cells are expensive because of the high cost of pure hole transportation materials. Besides, hole conductors such as the widely used spiro-OMeTAD are not only expensive but can cause moisture-induced degradation in PSCs, especially with hygroscopic dopants such as lithium bis(trifluoromethanesulfonyl)imide [8]. To overcome the lifetime-limiting problems with the organic hole transporters, HTM-free perovskite photovoltaics were proposed and reported by Etgar et al. [9]. HTM-free PSCs achieved efficiencies of about 10 % with gold as a back electrode [10–12]. The precious Au electrode also requires a high-vacuum and high-cost evaporation technique, thereby limiting its future application. Nowadays, the efficiency of the best PSCs is competitive with current commercial technologies, and they are potentially much cheaper. However, commercial solar cells must last 20–30 years with minimal degradation. The biggest challenge facing PSCs is long-term stability in a wide range of environments [7].

Low-cost nano-carbon can be an ideal material to substitute Au as a back contact in PSCs because its function is similar to that of Au. In the past decade, carbon nanomaterials have been demonstrated to be excellent counter-electrode candidates for use in dye-sensitized solar cells (DSSCs) owing to their various fascinating properties, including high electrical conductivity, thermal stability, good optical transparency, unique nanostructure, excellent electrocatalytic activity, low cost, and abundance [13–16]. Substantial gains have been made in the application of carbon nanomaterials in DSSCs; however, perovskite organic lead iodide is unstable at high temperatures or in some solvents [17]. Thus, the direct preparation of a carbon layer faces some problems. HTM-free PSCs with carbon contacts were first fabricated by infiltrating CH_3_NH_3_PbI_3_ or a mixed-cation 5-ammoniumvaleric (5-AVA) and MA perovskite (5-AVA)_*x*_(MA)_1−*x*_PbI_3_ into a high-temperature prefabricated monolithic device, which consists of four layers including TiO_2_ dense, TiO_2_ mesoporous, ZrO_2_ mesoporous, and carbon layers [18]. The (5-AVA)_*x*_(MA)_1−*x*_PbI_3_ perovskite device achieved a PCE of 12.8 %. The cells have shown promising stability under long-term light soaking and long-term heat exposure. But these devices employed complex structures and required processing temperatures of up to 400 °C to remove solvents and organic binders in the printed ZrO_2_ space and carbon black/graphite electrodes. The complicated fabrication and high-temperature processing increased material or energy consumption and limited their mass production and fabrication on a plastic substrate. Very recently, carbon counter electrodes were prepared using low-temperature (LT) processed (70–100 °C) carbon pastes and applied in HTM-free perovskite/TiO_2_ heterojunction solar cells to substitute noble metallic anodes. Under optimized conditions, a PCE in the range of 8.31–9.00 % has been demonstrated with these carbon counter electrodes [19, 20]. The HTM-free solar cell with the LT carbon counter electrode can have a much simpler structure, thereby reducing the cost and improving the overall stability of PSCs. However, the PSCs based on the LT carbon contact are poor in photovoltaic performance. Therefore, it is worth developing and improving the performance of HTM-free and carbon-based PSCs.

The perovskite layers with a well-defined grain structure, full surface coverage, and small surface roughness allow realization of an efficient solar cell [5, 21]. Therefore, various morphology control protocols including sequential deposition [4], thermal evaporation deposition [5], compositional engineering [22, 23], additive-assisted deposition [24, 25], solvent engineering [26–28], and intramolecular exchange processing [7] were investigated for high-quality perovskite absorbers. Jeon et al. [26] reported a solvent engineering method for highly uniform CH_3_NH_3_Pb(I_1−*x*_Br_*x*_)_3_ (*x* = 0.1–0.15) perovskite layers and high-efficiency organic HTM-based devices using a mixed solvent of γ-butyrolactone (GBL) and dimethyl sulfoxide (DMSO) followed by toluene (TO) drop-casting (DC). Although DMSO-based solvent engineering [26, 28] is a very potential experimental technique, the related processing involves the anfractuous coupling of fluid rheology, solvent evaporation, and molecular self-assembly, and the formation of high-quality perovskite film is the result of a complex dynamic process still under investigation. Furthermore, the morphology control methods have been mainly developed for organic HTM-based PSCs and rarely explored for HTM-free PSCs. However, the ideal morphology of HTM-free PSC also requires a uniform, highly crystalline, and high-coverage perovskite capping layer on the top of the mesoporous TiO_2_ [29].

In this work, we report a chlorobenzene (CB)-based solvent-assisted process toward synthesizing simple, stable, and efficient HTM-free PSCs with carbon counter electrodes. CH_3_NH_3_PbI_3_ absorber layers were synthesized on mesoporous TiO_2_ films via spin-coating the mixed solution of CH_3_NH_3_I, PbI_2_, GBL, and DMSO, followed by DC CB or TO while spinning. TO was studied as a DC solvent for comparison. The effects of the DC solvent and the annealing temperature on thin film morphology, crystal structure, and the solar cell performance were investigated. Through changing the DC solvent and optimizing the annealing temperature, extremely uniform and dense perovskite capping layers atop mesoporous TiO_2_ films were obtained, enabling the fabrication of remarkably improved HTM-free PSCs. The efficiency of simple structured HTM-free solar cells was high up to 11.42 %. The obtained carbon-based PSC devices have shown much more promising stability than the HTM devices.

## Experimental Section

### Materials

Unless specified, all materials were purchased from either Alfa Aesar or Sigma-Aldrich and used as received. Spiro-MeOTAD was purchased from Merck KGaA and Luminescence Technology Corp. MA iodide (MAI) CH_3_NH_3_I was synthesized according to a previous study [30].

### Device Fabrication

Fluorine-doped tin oxide-coated glass (Pilkington TEC 15) was patterned by etching with Zn powder and 2 M HCl. The etched substrate was then cleaned with surfactant and rinsed with acetone, ethanol, and deionized water. A 50-nm-thick compact TiO_2_ (*c*-TiO_2_) thin layer was synthesized by a procedure reported in our previous work [31]. The porous TiO_2_ (*p*-TiO_2_) layer was deposited by spin-coating at 5000 rpm for 30 s using a commercial TiO_2_ paste (Dyesol 18NR-T) diluted in ethanol (1:2.5 weight ratio) and consequently heated at 500 °C for 30 min. After cooling to room temperature, the as-prepared nanoporous TiO_2_ films were then dipped into a 40 mM TiCl_4_ aqueous solution for 30 min at 70 °C, dried at ambient atmosphere, and then sintered at 500 °C for 30 min.

CH_3_NH_3_PbI_3_ perovskite absorber layers were synthesized by modifying the solvent engineering method reported by Jeon et al. [26]. This was done in a glove box maintaining 10 % RH level. The synthesized CH_3_NH_3_I (0.1975 g) powders and lead iodide PbI_2_ (0.5785 g) were stirred in a mixture of GBL (700 μL) and DMSO (300 μL) at 60 °C for 12 h. The formed precursor solution was deposited onto *p*-TiO_2_/*c*-TiO_2_/FTO substrate by a successive two-step spin-coating process at 2000 rpm for 50 s and at 3500 rpm for 50 s, respectively. During the second step, anhydrous CB or TO was dripped onto the center of the sample 30 s prior to the end of the program. The perovskite precursor-coated substrate was heated and dried on a hot plate at a temperature of 50–140 °C for 10 min.

The carbon electrodes were prepared by doctor blade coating an LT conductive carbon ink (Shanghai Materwin New Materials Co., Ltd.) on the grown perovskite absorber, followed by drying at 100 °C for 30 min. For comparison, spiro-OMeTAD was deposited onto the perovskite absorber, and the metal cathode around 100 nm was deposited on the spiro-OMeTAD HTM layer by thermal evaporation under the base pressure of 6 × 10^−4^ Pa [31, 32].

### Characterization

The morphologies of the perovskite absorbers using different post-heating temperatures and various DC solvents were characterized by field emission scanning electron microscope (FESEM, Hitachi S4800). The structures of the formed perovskite absorbers were identified by X-ray diffractometer (XRD, Bruker D8 Davinci instrument, Cu Kα: *λ* = 0.15406 nm). Photocurrent density–voltage (*J–V*) measurements were performed using an AM 1.5 solar simulator equipped with a 1000 W xenon lamp (Model No. 91192, Oriel, USA). The solar simulator was calibrated using a standard silicon cell (Newport, USA). The light intensity was 100 mW cm^−2^ on the surface of the test cell. *J–V* curves were measured using a computer-controlled digital source meter (Keithley 2440) in the reverse direction. During device photovoltaic performance characterization, a metal aperture mask with an opening of about 0.09 cm^2^ was used.

## Results and Discussion

**Fig. 1:**
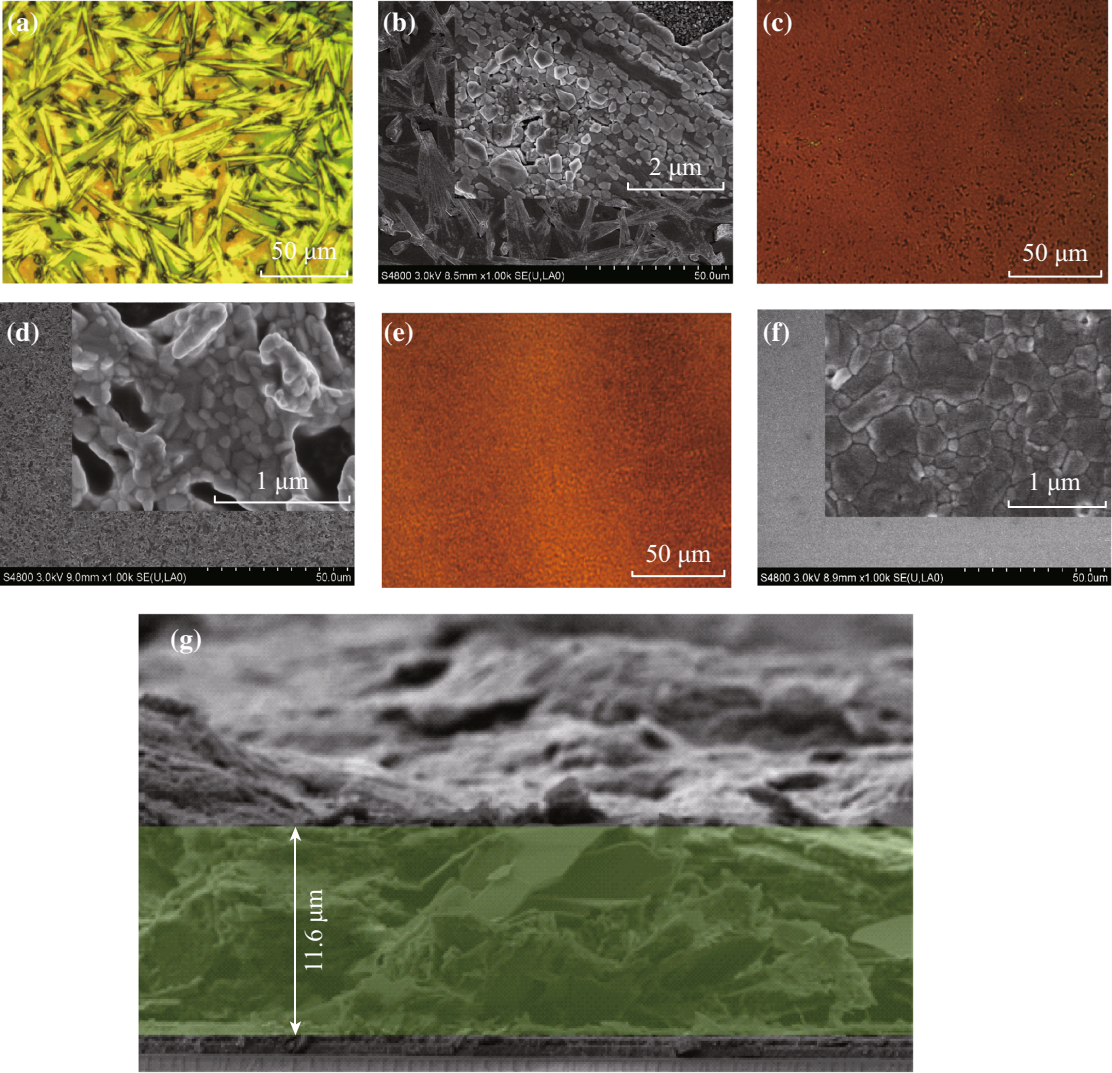
Optical and SEM images of CH_3_NH_3_PbI_3_ layers fabricated without drop-casting solvent (**a**, **b**) or with toluene (**c**, **d**) or chlorobenzene (**e**, **f**) as a drop-casting solvent. (**g**) Cross-section FESEM image of the PSCs

Initially, we investigated the effect of DC solvent on the morphology of CH_3_NH_3_PbI_3_ perovskite film grown on the *p*-TiO_2_ via the solvent engineering method. The post-heating temperature for the CH_3_NH_3_PbI_3_ absorber is 100 °C. Figure 1 shows the optical and SEM images of CH_3_NH_3_PbI_3_ layers fabricated without DC solvent or with CB or TO as a DC solvent. For the sample without using DC solvent, non-homogeneous perovskite film was formed, and rather large branch-like grains with a significant portion of the substrate (the *p*-TiO_2_ layer) being exposed without CH_3_NH_3_PbI_3_ coverage are seen in Fig. 1a, b, which is in accordance with previous observations [5, 33]. A higher magnification SEM image of these large branch-like grains reveals that the grain structure consists of crystals with sizes of 50–400 nm as shown in the inset of Fig. 1b. Crystals of the semiconductor perovskite CH_3_NH_3_PbI_3_ with a wide range of sizes were also reported when the perovskite was deposited by a single-step spin-coating from a solution of CH_3_NH_3_I and PbI_2_ in GBL or *N*,*N*-dimethylformamide (DMF) [4]. When TO was used as a DC solvent, the large branch-like grains disappeared in the optical image in Fig. 1c, and some smaller (35–140 nm) and more uniform crystals started to form, leading to reduced pinhole sizes and enhanced surface coverage of perovskites as shown in Fig. 1d. In contrast, when CB was used as a DC solvent, the mesoporous TiO_2_ layer was covered with interconnected crystals with a full surface coverage as clearly shown in the inset of Fig. 1f. The CH_3_NH_3_PbI_3_ films formed by the addition of CB are composed of sub-micron (100–550 nm)-sized grains, which are obviously larger than those in the absorber layer using TO as a DC solvent. For the former, the top surface exhibits a dense-grained morphology. The differences in the surface coverages of perovskite films on the *p*-TiO_2_ layer likely affect the device characteristics [29, 34]. The cross-section FESEM image of the PSCs is shown in Fig. 1g. The formed carbon film is very thick compared to the other functional layers in the device. The thickness of the carbon layer is about 11.6 μm. Its sheet resistance is about 14.6 Ω sq^−1^. The carbon film achieved good and tight adhesion to the underlayered CH_3_NH_3_PbI_3_ and provided complete coverage over the absorber.

**Fig. 2:**
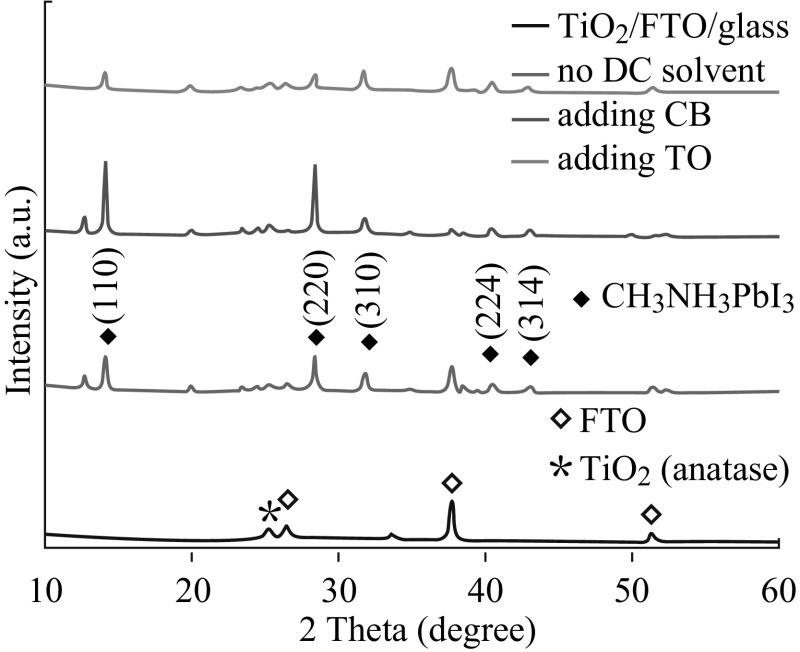
XRD spectra of CH_3_NH_3_PbI_3_ layers fabricated without drop-casting (DC) solvent or with chlorobenzene (CB) or toluene (TO) as a DC solvent

The XRD patterns of the above three samples are shown in Fig. 2. The red, blue, and purple curves indicate XRD spectra measured from CH_3_NH_3_PbI_3_ layers without DC solvent and with CB or TO as the DC solvent, respectively. Diffraction peaks observed at 14.02°, 28.32°, 31.76°, 40.46°, and 43.02° correspond to the reflections from (110), (220), (310), (224), and (314) crystal planes of the tetragonal perovskite structure, respectively [35, 36]. It was observed that compared to the case of the pristine absorber without DC solvent modification, intense diffraction peaks on both the (110) and (220) facets became significantly enhanced with the addition of CB, indicative of the improvements in the crystalline property of the CH_3_NH_3_PbI_3_ film. Also, the optical and SEM images indicate the enlarged crystalline domains in the lateral direction with the morphology evolution from branch like to plate like (Fig. 1). However, compared to the case without DC solvent, both (110) and (220) peaks became obviously reduced with the addition of TO, which can be attributed to the reduced quality of the perovskite crystals in the latter as shown in the inset of Fig. 1b, d.

**Fig. 3:**
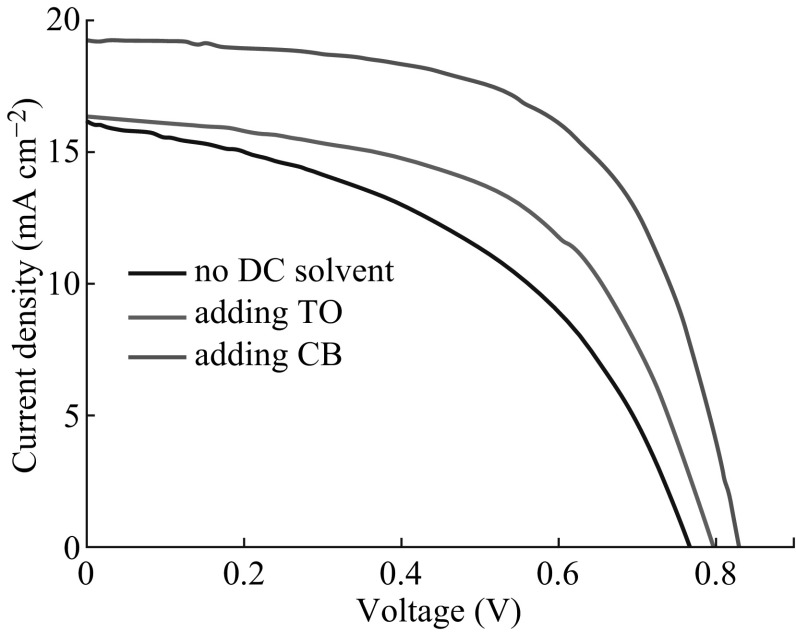
*J–V* *curves* for carbon-based solar cells with CH_3_NH_3_PbI_3_ layers fabricated without drop-casting (DC) solvent or with chlorobenzene (CB) or toluene (TO) as a DC solvent

To investigate the effect of the additional solvent on the HTM-free device performance, carbon electrode-based PSCs were fabricated without DC solvent or with CB or TO as a DC solvent. The post-heating temperature for the CH_3_NH_3_PbI_3_ absorber is 100 °C. For each device fabrication condition, 8–12 PSCs were fabricated in an identical manner. The average device characteristics are shown in Fig. 3. The corresponding photovoltaic parameters including short circuit current density (*J*_SC_), open circuit voltage (*V*_OC_), PCE, and fill factor (FF) are listed in Table 1. As shown in Fig. 3 and Table 1, the PSC with the pristine perovskite film showed a *J*_SC_ of 16.10 mA cm^−2^, a *V*_OC_ of 0.77 V, and an FF of 0.46, therefore an overall PCE of 5.70 %. Using TO droplets, the fabricated device showed an enhanced *V*_OC_ of 0.80 V, an FF of 0.55, and a similar *J*_SC_ of 16.31 mA cm^−2^, thus an improved PCE of 7.17 %, leading to about 26 % enhancement of PCE. Remarkably, through the introduction of CB into the perovskite precursor layer, the PCE increased to 9.73 %. The introduction of CB droplets resulted in a simultaneous improvement of all the device parameters, e.g., *J*_SC_ increased from 16.10 to 19.21 mA cm^−2^, *V*_OC_ from 0.77 to 0.83 V, and FF from 0.46 to 0.61, and thus about 71 % enhancement of PCE in the device.

**Table 1:** Photovoltaic performance of solar cells with CH_3_NH_3_PbI_3_ layers fabricated without assisted solvent or with chlorobenzene or toluene as an assisted solvent

Samples	*J*_SC_ (mA cm^−2^)	*V*_OC_ (V)	FF	PCE (%)	*R*_S_ (Ω cm^2^)	*R*_SH_ (Ω cm^2^)
No drop-casting solvent	16.10	0.77	0.46	5.70	129	1996
Adding toluene	16.31	0.80	0.55	7.17	121	4601
Adding chlorobenzene	19.21	0.83	0.61	9.73	73	11,175

The morphology and the crystal structure evolution of the perovskite film with CB droplet treatment shown in Figs. 1 and 2 could be attributed to the formation of CH_3_NH_3_I–PbI_2_–DMSO intermediate phase during spin-coating from MAI, PbI_2_, and DMSO [26]. This process can be regarded as the transformation of PbI_2_–DMSO–MAI into MAPbI_3_, similar to the case with a TO DC. Jeon et al. [26] deposited high-quality CH_3_NH_3_Pb(I_1−*x*_Br_*x*_)_3_ (*x* = 0.1–0.15) perovskite layers through the use of a combination of DMSO/GBL followed by a TO drip. They found that the spin-coated PbI_2_–DMSO–MAI intermediate phase possessed an extremely uniform and flat morphology, the intermediate phase was partly transformed into perovskite phases at 100 °C, and the complete transformation into CH_3_NH_3_Pb(I_1−*x*_Br_*x*_)_3_ took place at an annealing temperature of 130 °C. But, from Fig. 2, pure perovskite phases were obtained at 100 °C for treatment with both CB and TO in our synthesis. Our results are in accordance with those reported in [27]. High-quality CH_3_NH_3_PbI_3_ films were obtained for a fast deposition–crystallization procedure at 100 °C with the assistance of CB in the latter.

**Fig. 4:**
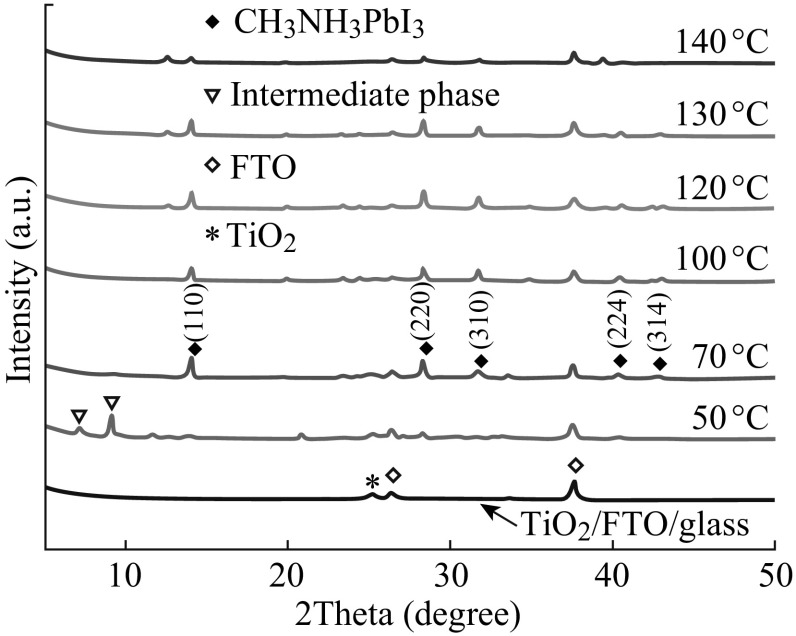
XRD patterns of the perovskite films grown on the *p*-TiO_2_ via chlorobenzene drop-casting at different annealing temperatures of 50–140 °C

Thermal energy directly dominates the thermodynamics of the crystalline perovskite film formation in solution processes. Controlling the thermal annealing process for the perovskite precursor film is a key to achieving high performance [17, 34]. In this work, we found that annealing temperature plays an important role in the quality of the perovskite absorber. Figure 4 shows the XRD patterns of the perovskite films grown on the *p*-TiO_2_ via the solvent-assisted method with different annealing temperatures. The CH_3_NH_3_PbI_3_ layers were fabricated with CB treatment. The post-heating temperature was changed from 50 to 140 °C. When a low annealing temperature of 50 °C was used, XRD peaks at the low angles of 7.21° and 9.17° can be attributed to the MAI–PbI_2_–DMSO intermediate phase in the film [26]. When the annealing temperature was increased to 70 °C, only a very weak peak at 9.17° was observed. When the annealing temperature was equal to or greater than 100 °C, XRD peaks at the low angles disappeared completely. Besides, when the annealing temperature was equal to or greater than 70 °C, peaks at 14.02°, 28.32°, 31.76°, 40.46°, and 43.02°, corresponding to the reflections from (110), (220), (310), (224), and (314) crystal planes of the tetragonal perovskite structure, respectively [35, 36], were detected. The XRD results indicated that the MAI–PbI_2_–DMSO intermediate phase was fully transformed into perovskite phases at 100 °C for our pure halide material system with CB DC. Moreover, a small peak at 12.66°, which is associated with the PbI_2_ film, is visible in the XRD pattern for the absorber formed at 120–140 °C. The intensity of this PbI_2_ phase peak increased with the temperature rising from 120 to 140 °C, while the perovskite phase peaks decreased with the temperature increase. It is worth mentioning that annealing at 120 °C brought about majority of the conversion of the precursor film to the active perovskite phase and retention of a small fraction of PbI_2_, as exhibited by the small peak at 12.66°. Although this peak expresses incomplete conversion of the PbI_2_–DMSO–MAI film to active perovskite phase, the presence of a little residual PbI_2_ has been found to be advantageous for device performance [37, 38], likely due to passivation of surface and grain boundary states [37].

**Fig. 5:**
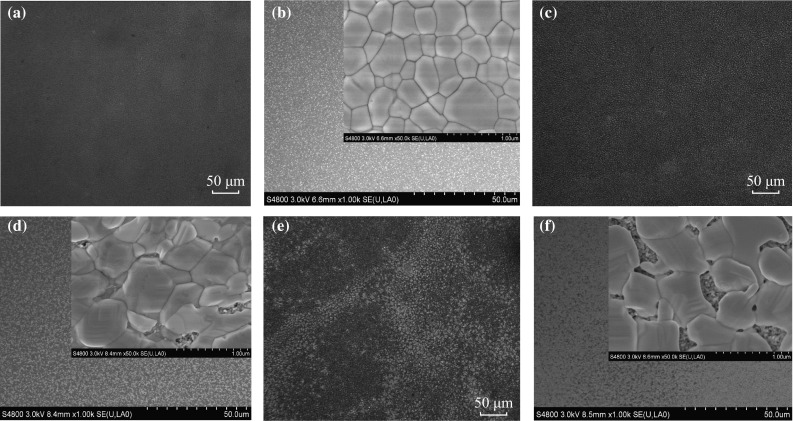
Optical and SEM images of the perovskite films grown on the *p*-TiO_2_ via chlorobenzene drop-casting at different annealing temperatures: **a**, **b** 120 °C, **c**, **d** 130 °C, and **e**, **f** 140 °C

Figure 5 shows the optical and SEM images of the perovskite films grown on the *p*-TiO_2_ via the solvent-assisted method with various annealing temperatures of 120–140 °C and CB DC. Annealing at 120 °C resulted in the formation of plate-like, uniform, and well-crystallized perovskite layer. The entire layer is composed of homogeneous, interconnected, and perfectly crystallized crystals with 100 % surface coverage atop the *p*-TiO_2_ shown in the inset of Fig. 5b. The perfect absorber layer is composed of sub-micron (250–750 nm)-sized grains, which are more uniform and larger than those in the CH_3_NH_3_PbI_3_ film formed with CB treatment and an annealing temperature of 100 °C shown in Fig. 1e, f. When the annealing temperature increased further from 120 °C, however, the quality of the resulted absorber degraded and the surface coverage of perovskites decreased. At 130 °C, the grown absorber layer is composed of sub-micron-sized grains with some portion of the *p*-TiO_2_ layer being exposed without CH_3_NH_3_PbI_3_ coverage as clearly shown in the inset of Fig. 5d. Annealing at 140 °C led to larger pinhole sizes and lower surface coverage of perovskites as evidenced in the inset of Fig. 5f.

**Fig. 6:**
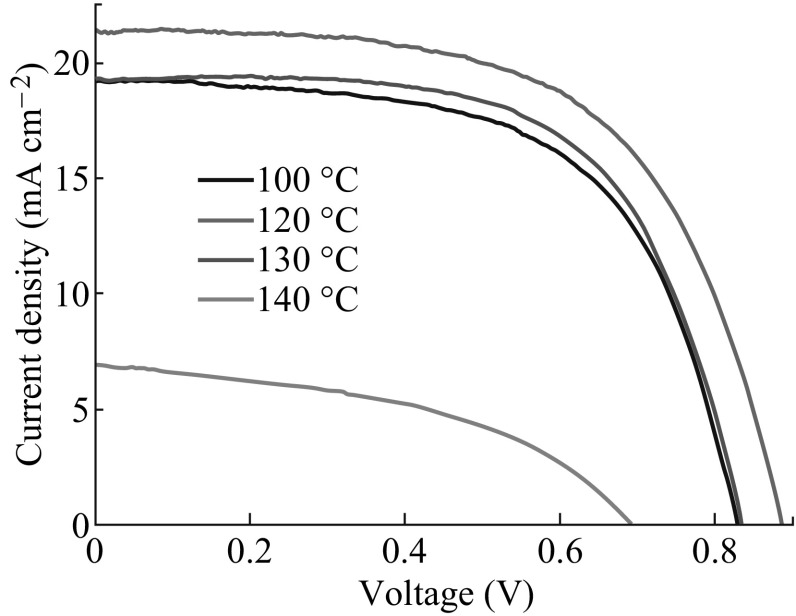
*J–V* *curves* for the solar cells with CH_3_NH_3_PbI_3_ layers fabricated via chlorobenzene drop-casting at different annealing temperatures of 120–140 °C

**Fig. 7:**
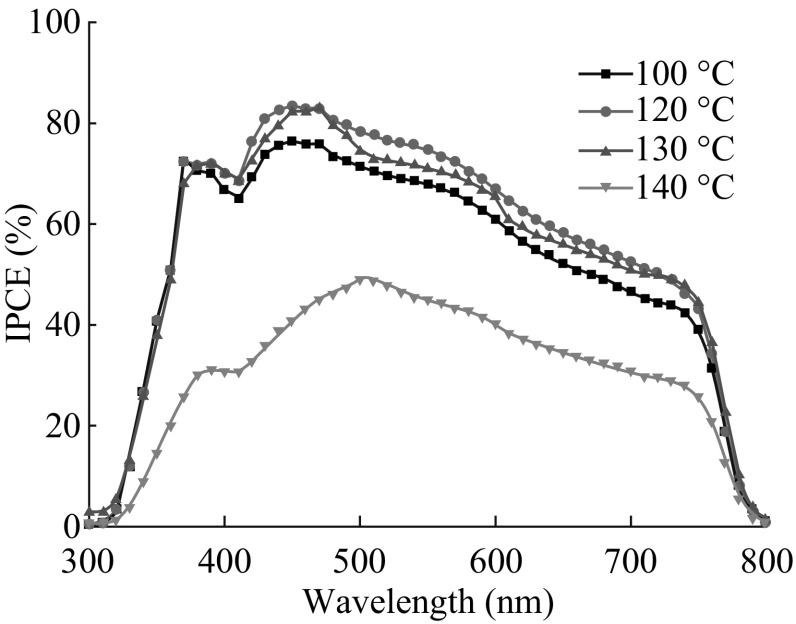
IPCE spectra of the solar cells with CH_3_NH_3_PbI_3_ layers fabricated via chlorobenzene drop-casting at different annealing temperatures of 120–140 °C

To investigate the effect of annealing temperature on the HTM-free device performance, PSCs were fabricated with CH_3_NH_3_PbI_3_ absorbers obtained via DC CB and annealing at different temperatures from 100 to 140 °C. For each temperature, 8–12 PSCs were fabricated in an identical manner. The average device characteristics are presented in Fig. 6, the corresponding photovoltaic parameters of which are summarized in Table 2. A clear correlation was observed between the annealing temperature of the perovskite and the photovoltaic performance of the device. Samples fabricated at 120 °C exhibited the highest PCE, 11.44 %, as a result of the *J*_SC_, *V*_OC_, and FF, 21.43 mA cm^−2^, 0.89 V, and 0.60, respectively. Devices grown at 130 °C also exhibited a quite high PCE of 10.21 %, as a result of the *J*_SC_, *V*_OC_, and FF, 19.29 mA cm^−2^, 0.84 V, and 0.63, respectively. But, as the annealing temperature was increased further above 130 °C, the *J*_SC_, *V*_OC_, and FF values considerably decreased, leading to a dramatic fall in PCE. The best photovoltaic performance of the device with 120 °C can be mainly associated with the highest crystalline, morphological, and surface coverage quality of its absorber shown in Figs. 4 and 5. These experimental observations are consistent with the results reported in [17, 34]. The corresponding monochromatic incident photon-to-electron conversion efficiency (IPCE) spectra of these four devices are shown in Fig. 7. Aside from the PSCs with an annealing temperature of 140 °C, the other three exhibited broad and efficient photoelectric conversion covering the range of visible light. Especially, the devices with 120 and 130 °C achieved much higher IPCEs than the cell with 140 °C over the whole spectral range between 300 and 800 nm, matching the difference in photocurrents obtained for these devices shown in Fig. 6 and Table 2.

**Table 2:** Photovoltaic performance of solar cells with CH_3_NH_3_PbI_3_ layers fabricated via assistance of chlorobenzene at different annealing temperatures of 120–140 °C

Annealing *T* (°C)	*J*_SC_ (mA cm^−2^)	*V*_OC_ (V)	FF (%)	PCE (%)	*R*_S_ (Ω cm^2^)	*R*_SH_ (Ω cm^2^)
100	19.21	0.83	0.61	9.73	73	11,175
120	21.43	0.89	0.60	11.44	74	18,808
130	19.29	0.84	0.63	10.21	70	22,404
140	6.93	0.69	0.45	2.15	293	3495

Moreover, the series resistance (*R*_S_) and the shunt resistance (*R*_SH_) were estimated from the *J–V* characteristics shown in Figs. 3 and 6. The *R*_S_ and *R*_SH_ data are summarized in Tables 1 and 2. For solar cells, maintaining the *R*_S_ as low as possible is vitally important because large *R*_S_ will decrease *J*_SC_, *V*_OC_, and FF and consequently PCEs [31, 39, 40]. For the devices with 100 °C, the *R*_S_ from the PSC with the addition of CB is 73 Ω cm^2^, which is smaller than 121 or 129 Ω cm^2^ from the device with the introduction of TO or without DC solvent, respectively. In the case of the CB treatment, when the temperature was increased from 100 to 130 °C, the *R*_S_ remained at the low values of about 70 Ω cm^2^. But, when the temperature was increased up to 140 °C, the *R*_S_ of the device increased to 293 Ω cm^2^. The low *R*_S_ from the device with the addition of CB and the annealing temperatures of 100–130 °C is due to small contact resistance and low bulk resistance of the high-quality perovskite layer, indicating that high currents can flow through the cell at low applied voltages [39]. Typically, the shunt resistance (*R*_SH_) is due to p–n junction non-idealities and impurities near the junction, which bring about partial shorting of the junction, especially near cell edges. The *R*_SH_ must be higher to avoid current loss at the junction [40], dwindling the photocurrent and consequently the solar cell performance. It has been diffusely reported that the pinholes formed in the solution-processed CH_3_NH_3_PbI_3−*x*_Cl_*x*_ absorbers can cause direct contact between the *p*-type spiro-OMeTAD and the TiO_2_ compact layer, leading to a shunting path that is probably partially responsible for the low FF and open circuit voltage in devices [5, 17]. In the case of our HTM-free PSCs with 100 °C shown in Table 1, the pinhole or voids in the capping layer on the *p*-TiO_2_ layer caused the least *R*_SH_, resulting in the lowest FF and *V*_OC_ in the device with pristine absorber, and induced the second least *R*_SH_, leading to the second lowest FF and *V*_OC_ in the device via dropwise TO application. The smoother perovskite layer due to introducing TO or CB into the spin-coated perovskite precursor layer from the mixed solution of CH_3_NH_3_I, PbI_2_, GBL, and DMSO decreased the series resistance and increased the shunt resistance of cell, resulting in the higher photovoltaic effects (Table 1). In the case of different annealing temperatures with the CB treatment shown in Table 2, as the temperature was increased from 100 to 130 °C, the shunt *R*_SH_ had the higher and higher resistive value, but, when the temperature was increased up to 140 °C, the pinhole or voids in the capping layer on the *p*-TiO_2_ layer caused the least *R*_SH_, resulting in the lowest FF and *V*_OC_ in the device. Therefore, the improved device performance can be associated with the evolved and promoted morphological and crystalline properties of CH_3_NH_3_PbI_3_ film by use of CB or TO droplets and optimized annealing temperature, as exhibited by optical, SEM, and XRD measurements in Figs. 1, 2, 4, and 5. The improved device performance unambiguously verified the significance of CB droplets and annealing temperature on PSC optimization. The possible mechanisms for improving the quality of the perovskite absorber layer with DC solvent and enhancing the resulted PSC performance are further explored below.

The boiling points of the traditional solvents used for growing perovskite absorbers are relatively high, having the values of 153 and 204–205 °C for *N*,*N*-dimethylmethanamide (DMF) and GBL, respectively. Such relatively high boiling points can result in the formation of large-area thickness variations or shrinkage/dewetting of the casting precursor solution due to the relatively prolonged drying times of solution-coated films. In our work, the proposed solvent- assisted technology involved spin-coating the mixed solution of CH_3_NH_3_I, PbI_2_, and GBL/DMSO, followed by DC CB or TO while spinning. TO (boiling point 111 °C, vapor pressure 22 mmHg) and CB (boiling point 132 °C, vapor pressure 11.8 mmHg) have much lower boiling point and tremendously higher vapor pressure than DMSO (boiling point 189 °C, vapor pressure 0.6 mmHg) and GBL (boiling point 204–205 °C, vapor pressure 1.5 mmHg) at room temperature. Considering the physical properties of the solvents, at the beginning stage of the spin-coating process, the film was composed of MAI and PbI_2_ dissolved in the DMSO/GBL solvent mixture, and the precursor film continued to thin because liquid flowed radially owing to the action of centrifugal force, and solvent evaporation was neglected, while in the intermediate stage, the composition of the film was concentrated by the evaporation of GBL due to its higher vapor pressure than DMSO. Then, the introduction of TO or CB droplets with very higher vapor pressure during the second spinning stage induced the immediate freezing of the constituents on spinning and the rapid formation of the MAI–PbI_2_–DMSO phase, producing a full and even precursor layer. The use of DMSO helped retard the rapid reaction between the inorganic PbI_2_ and CH_3_NH_3_I components of perovskite during the evaporation of solvents in the spin-coating process because DMSO has a stronger coordination ability with PbI_2_ than that of the usually used DMF [41]. At last, the greatly homogeneous and flat precursor film was converted into a pure crystalline CH_3_NH_3_PbI_3_ perovskite layer after annealing at 100 °C. As displayed in Figs. 1, 2, and 3 and Table 1, CH_3_NH_3_PbI_3_ films via the dropwise CB application showed better morphological, crystalline, and photovoltaic properties than the perovskite layers using TO droplet treatment. For a highly volatile solvent such as TO, one can expect to have very significant evaporation during the spin-off stage. The strong evaporation of the casting TO solvent possibly affects fluid rheology and vice versa [42, 43], tending to give relatively rough precursor films. In contrast, CB has a lower evaporation rate due to its relatively high boiling point and lower vapor pressure compared to those of TO, which should be a benefit to form a flatter, smoother, and more uniform MAI–PbI_2_–DMSO intermediate phase layer. The higher quality intermediate phase layer resulted in more highly crystalline and more homogeneous CH_3_NH_3_PbI_3_ perovskite capping layer atop the *mp*-TiO_2_ after the heat treatment at 100 °C, which caused the lower series resistance, higher shunt resistance, and better photovoltaic performance in the PSC (Fig. 3; Table 1). The fabricated HTM-free PSCs based on CB treatment demonstrated a PCE of 9.73 %, which is 71 and 36 % higher than those of the control device fabricated from the pristine perovskite film or via TO droplet treatment, respectively. The improved device performance unambiguously verified the significance of CB droplets on HTM-free PSC optimization. After further optimizing the annealing temperature, the HTM-free PSCs based on the incorporation of CB achieved a PCE of 11.44 %.

**Fig. 8:**
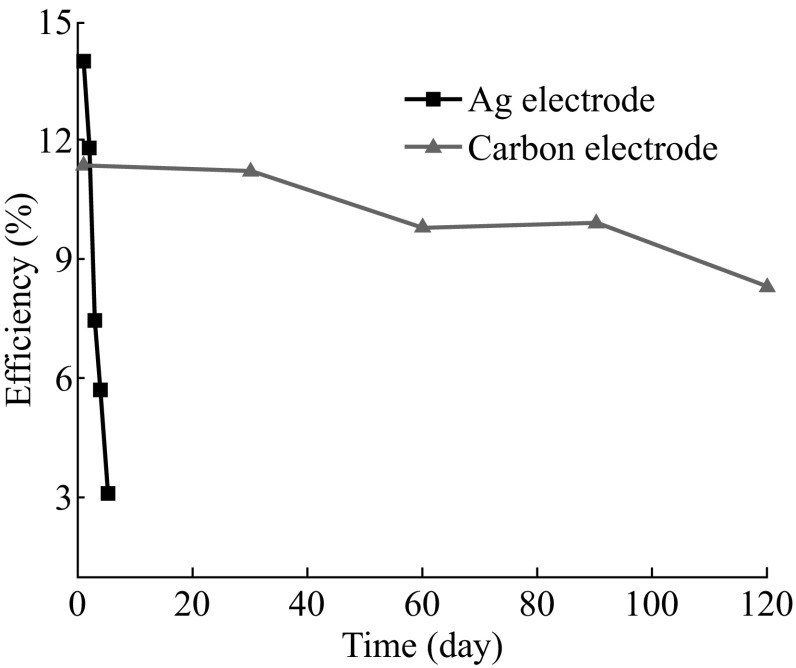
Stability profile (PCE) of non-sealed FTO/*c*-TiO_2_/*p*-TiO_2_/CH_3_NH_3_PbI_3_/spiro-MeOTAD/Ag (*squares*) and FTO/*c*-TiO_2_/*p*-TiO_2_/CH_3_NH_3_PbI_3_/carbon (*triangles*) perovskite solar cells. The devices were kept in a dry cabinet of electronics without nitrogen (10 % RH, room temperature)

Moreover, the fabricated HTM-free solar cells are significantly more stable than the spiro-OMeTAD HTM-based devices with Ag electrode shown in Fig. 8. The PCE of carbon-based device was over 8 % after 120 days, while the efficiency of the HTM-based cell dropped to 3 % only after 5 days. We hope that our findings can provide a better understanding of the crystalline perovskite film formation in solvent-assisted processes and make a contribution to the development of low-cost and stable PSCs.

## Conclusions

The type of the DC solvent and annealing temperature employed during the preparation of CH_3_NH_3_PbI_3_ films via a solvent-assisted process have a considerable impact on the resulting absorber morphologies, crystalline structures, and device photovoltaic performance. The advantages using CB as an assisted solvent become apparent upon comparing the *J–V* curves and PCEs of the carbon-based HTM-free CH_3_NH_3_PbI_3_ devices prepared using CB or TO as a DC solvent or without DC solvent. The CH_3_NH_3_PbI_3_ films grown using CB show better morphological, surface coverage, and crystalline properties than the perovskite layers formed by the addition of TO during processing under the same conditions. The pristine perovskite layers without using DC solvent exhibit the even poorer morphological and crystalline properties. The HTM-free devices based on CH_3_NH_3_PbI_3_ fabricated by incorporation of CB show superior PCEs. The effects of the annealing temperature on CH_3_NH_3_PbI_3_ film morphology, crystal structure, and the solar cell performance were also investigated for the CB-assisted process. High-efficiency carbon-based HTM-free solar cells with a PCE of 11.44 % were produced using an optimized annealing temperature of 120 °C. This work provides an effective protocol for fabricating efficient, simple, stable, and low-cost inorganic–organic hybrid heterojunction solar cells.
